# T cell migration in microchannels densely packed with T cells

**DOI:** 10.1038/s41598-019-43569-w

**Published:** 2019-05-10

**Authors:** HyoungJun Park, Junsang Doh

**Affiliations:** 1 Department of Mechanical Engineering, Pohang University of Science and Technology (POSTECH) San 31, Hyoja-dong, Nam-Gu, Pohang, Gyeongbuk 37673 Korea; 2 School of Interdisciplinary Bioscience and Bioengineering (I-Bio), Pohang University of Science and Technology (POSTECH) San 31, Hyoja-dong, Nam-Gu, Pohang, Gyeongbuk 37673 Korea; 3 Department of Materials Science and Engineering, Seoul National University, 1 Gwanak-ro, Gwanak-gu, Seoul, 08826 South Korea

**Keywords:** Amoeboid migration, Biomedical engineering

## Abstract

T cells migrate diverse microenvironments of the body to mount antigen-specific immune responses. T cell activation, a key initial process for antigen-specific immune responses, occur in secondary lymphoid organs such as spleens and lymph nodes where high density of T cells migrates rapidly through the reticular networks formed by stromal cells. *In vitro* model system recapitulating key characteristics of secondary lymphoid organs, confined spaces densely packed with rapidly migrating cells, would be useful to investigate mechanisms of T cell migration. In this study, we devised a method to fabricate microchannels densely packed with T cells. Microchannel arrays with fixed height (4 μm) and length (1.5 mm) and various widths (15~80 μm) were fabricated in between trapezoid-shaped reservoirs that facilitated T cell sedimentation near microchannel entries. Microchannel surface chemistry and filling time were optimized to achieve high packing density (0.89) of T cell filling within microchannels. Particle image velocimetry (PIV) analysis method was employed to extract velocity field of microchannels densely packed with T cells. Using velocity field information, various motility parameters were further evaluated to quantitatively assess the effects of microchannel width and media tonicity on T cell motility within cell dense microenvironments.

## Introduction

Rapid trafficking to specific organs/tissues and robust motility within diverse tissue microenvironments are critical characteristics of T cells as orchestrators of antigen specific immune responses^1,2^. For immune surveillance, T cells circulate secondary lymphoid organs such as spleens and lymph nodes (LNs) where information of potentially harmful antigens is collected and presented by antigen presenting cells (APCs)^3,4^. T cell activation by T cell-APC interactions in secondary lymphoid organs is a key event for the initiation of antigen-specific immune responses. Activated T cells undergo clonal expansion, and traffic to effector sites to mount antigen-specific immune responses.

For efficient antigen-specific immune responses, T cell trafficking in blood vessels and migration in interstitial spaces of various tissues/organs need to be optimally regulated. Intravital imaging enabled us to directly observe stunning T cell dynamics under various physiological/pathological settings and provided us numerous information on T cell motility in diverse microenvironments^5–9^. However, mechanistic study using live animals is technically challenging, thus *in vitro* model system recapitulating key features of *in vivo* microenvironments has been developed. For example, parallel flow chambers mimicking blood vessel microenvironments have been widely used to study dynamic T cell-endothelial cell interactions under flow^10,11^. Collagen gels have been used to study 3D interstitial migration of T cells^12,13^. Based on the fact that leukocytes, including dendritic cells and T cells, in 3D interstitial spaces squeeze through porous spaces and exhibit amoeboid migration with no degradation of extracellular matrixes (ECMs)^12–15^, straight microchannels recapitulating ‘confinement’ as a key characteristics of 3D interstitial spaces have been developed and used. For example, dendritic cell migration in peripheral tissue^16^, T cell motility in interstitial spaces regulated by myosin proteins^17,18^, and leukocyte chemotactic responses^19^ were studied using microchannel devices. This simple model has been extremely useful for mechanistic study because motility of leukocytes in microchannels was similar to that of *in vivo* interstitial spaces, whereas cell manipulation and data acquisition/processing are much easier than intravital imaging. So far, microchannel experiments have been primarily conducted to observe single leukocyte migration within microchannels using low density of leukocytes, which mimics leukocyte migration in peripheral tissues where leukocytes are sparsely distributed. However, this model may not fully recapitulate cell dense microenvironments in secondary lymphoid organs such as spleens and LNs, where high density of lymphocytes forms segregated compartments and exerts rapid motility through the reticular network generated by stromal cells within the compartments^20,21^. In addition to leukocyte interstitial migration study, microchannels have been widely used to study the migration of various types of cells in confined 3D microenvironments. For example, mechanisms of cell migration under confinement^22–24^, cancer cell invasion dynamics^25,26^, and confinement-mediated nuclear envelope rupture and repair were studied^27,28^. However, all the aforementioned studies have primarily focused on single cell migration within microchannel.

In this study, we fabricated microchannels with various widths, and developed a method to fill T cells in the microchannels with high packing density (~0.9). Particle image velocimetry (PIV) technique was applied to extract velocity field information of T cells within the microchannels. Using PIV data, other kinematic parameters such as order parameter, which measures directional orientation with respect to microchannel walls, and vorticity, which represents local rotation, were calculated. Pharmachological inhibitors widely used *in vitro* cell biology study cannot be utilized in this experimental setting because most inhibitors were absorbed by T cells locating near microchannel entries. Instead, we adjusted tonicity of media to study the role of cell membrane tension on T cell migration within microchannels densely packed with T cells.

## Results and Discussions

### T cell filling in microchannels

**Figure 1:**
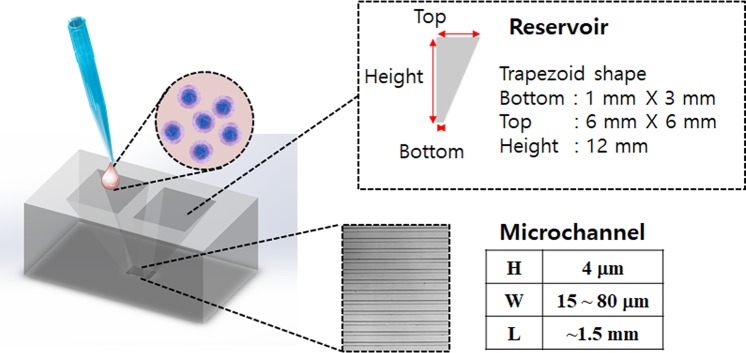
Schematic illustration of microchannels densely packed with T cells. PDMS microchannel arrays with trapezoid reservoirs located at each microchannel end were fabricated. Height (H) and length (L) of microchannels were fixed to 4 μm and 1.5 mm, respectively, whereas width (W) of microchannels were varied from 15 to 80 μm.

Microchannels with various channel widths (15~80 μm) and fixed height (4 μm) and length (~1.5 mm) were fabricated in between two reservoirs (Fig. 1). Each device contained an array of microchannels with one microchannel width, thus different devices were used for microchannels with difference channel widths. Media containing T cells (10^7^ cells/mL) was applied to both reservoirs. The trapezoid shaped reservoir guided sedimentation of T cells toward the entrance of microchannels. T cells sedimented down to the bottoms gradually migrated into the microchannels.

**Figure 2:**
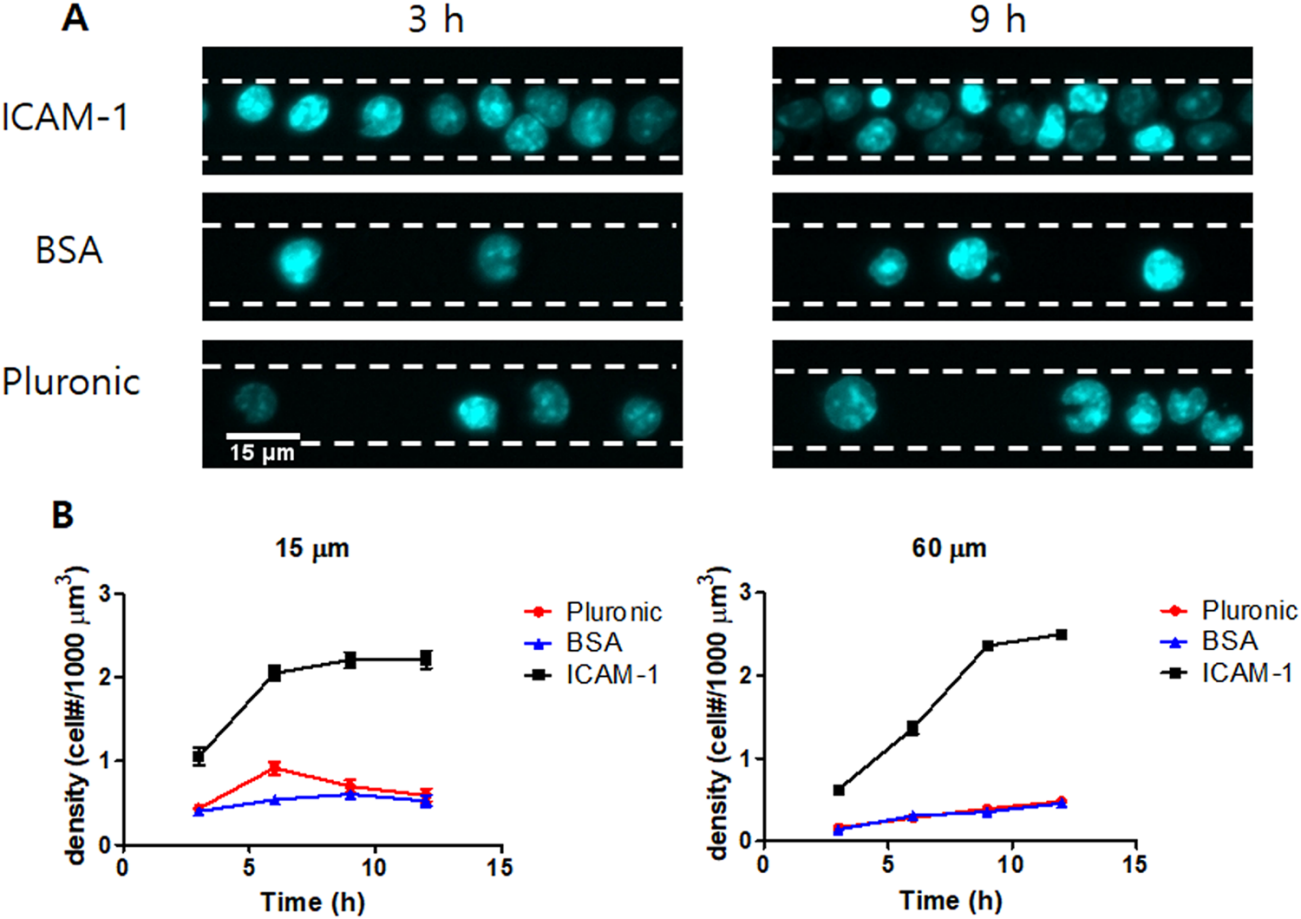
Kinetics of T cell filling in microchannels. Microchannels coated with ICAM-1, BSA, and Pluronic F-127 were filled with T cells. Kinetics of T cell filling in microchannels with two different widths, W = 15 and 60 μm, was assessed by acquiring fluorescence images of Hoechst 33342, which labels nucleus of T cells, up to 12 h with 3 h intervals. (**A**) Snapshots of T cell nucleus in microchannels (W = 15 μm) coated with various materials acquired at 3 and 9 h after filling. White dashed lines: microchannel walls. (**B**) Kinetics of T cell filling in microchannels coated with various materials. *n* = 32 for each condition (15 μm) and *n* = 22 for each condition (60 μm). Error bars: standard error of the mean (s.e.m.). Data are representative of four independent experiments.

To assess how microchannel surfaces affects T cell filling, the microchannels were coated with intercellular adhesion molecule 1 (ICAM-1), which is a ligand of T cell integrin lymphocyte function-associated antigen 1 (LFA-1)^29^, or cell-repellent materials such as bovine serum albumin (BSA) and pluronic^30^. Kinetics of T cell filling was monitored by measuring number of cells/unit area in microchannels at different time points after seeding. T cells labeled with Hoechst 33342, which stains nuclei of live cells, were seeded in microchannels. Fluorescence images of Hoechst 33342 were acquired every 3 h after seeding at the center region of microchannel, 600~900 μm apart from entry (Fig. 2A), to measure density of T cells in microchannels at different time points (Fig. 2B). Overall, T cell density in ICAM-1-coated microchannels was substantially higher than that of BSA- or pluronic-coated microchannels for all experimental conditions. T cells in BSA- or pluronic-coated microchannels only partially filled microchannels even 12 h after seeding. In contrast, T cells in ICAM-1-coated microchannels densely filled microchannels with density of ~2.5 × 10^−3^ cells/μm^3^, the same order of magnitude as the T cell density in lymph nodes^31^, within 9 h of seeding regardless of microchannel widths. The highest packing density of T cells in ICAM-1 coated microchannels was approximately 0.89 (calculated using the diameter of T cells = 8.80 ± 0.06 μm), comparable to that of hexagonal close packing in 2D (0.91), meaning T cells were tightly packed within the microchannels. Considering T cells within BSA- or pluronic-coated microchannels migrated well, ICAM-1 coating is required for high density filling of T cells in microchannels, but may not be necessary for T cell motility within microchannels. Based on this kinetics of microchannel filling, the rest of the experiments were performed using ICAM-1 coated microchannels by observing T cells 9~12 h after seeding. Indeed, ICAM-1-coated microchannels were used to recapitulate T cell migration in lymph nodes because ICAM-1 is ubiquitously expressed in cells comprising lymph nodes^17,18^.

### PIV analysis of T cell migration in microchannels densely packed with T cells

**Figure 3:**
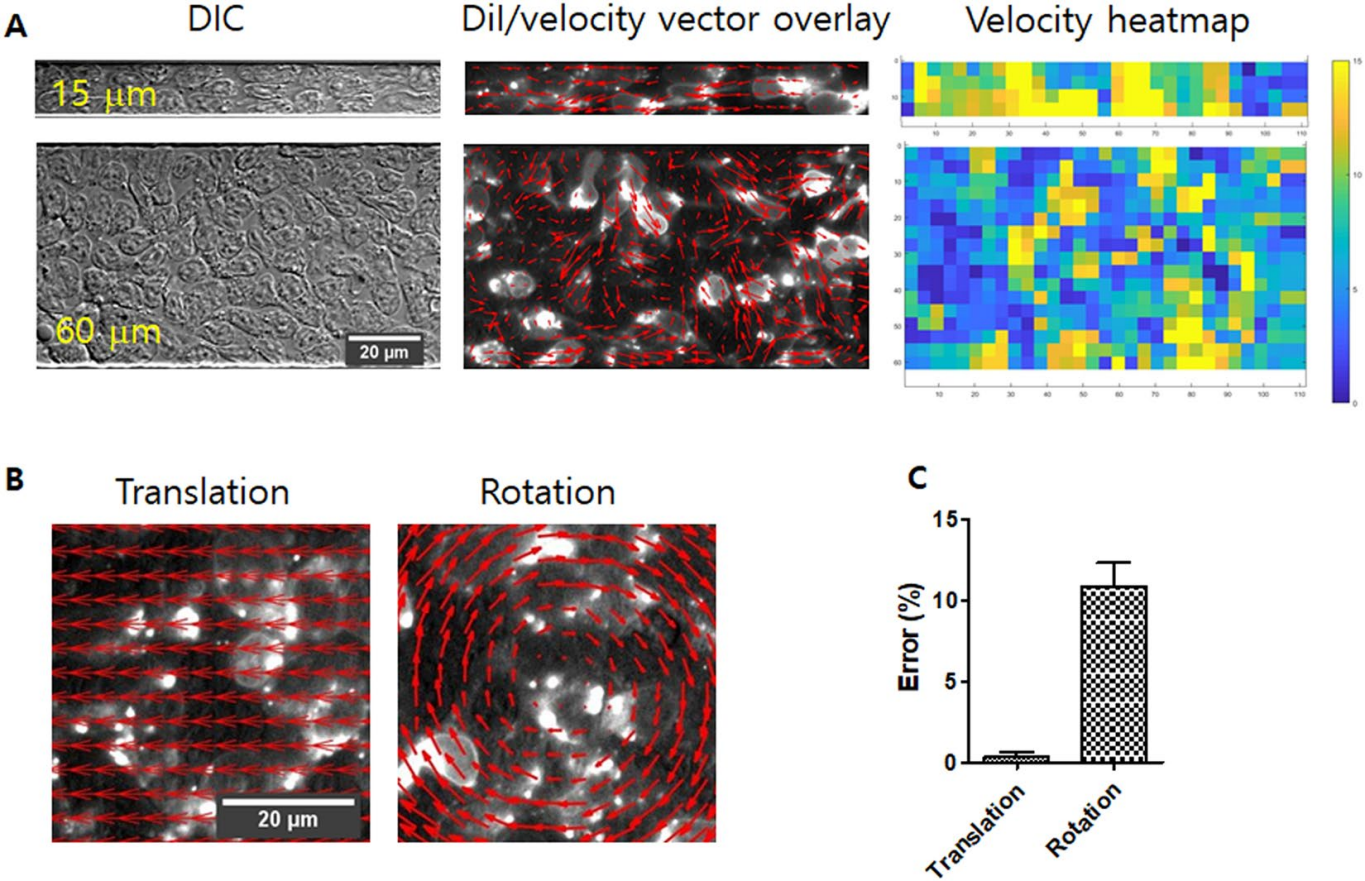
PIV analysis of T cell migration in microchannels densely packed with T cells. (**A**) Snapshot images of densely packed T cells in microchannels. DIC images (left), DiI labeled fluorescence images overlayed with velocity vectors (red arrows) obtained by PIV analysis (middle), and velocity heatmap (right) were shown for microchannels with W = 15 and 60 μm. (**B**) Snapshot images of synthetic movies of translation and rotation overlayed with velocity vectors obtained by PIV. (**C**) Errors of PIV analysis of synthetic movies shown in (**B**). *n* = 4 for translation, and *n* = 5 for rotation. Error bars: s.e.m.

Since tracking individual T cells in microchannels densely packed with T cells was technically challenging, we labeled T cells with 0.3% vybrant DiI, which generates speckle patterns due to non-homogeneous labeling, and indirectly tracked T cells by performing PIV analysis (Fig. 3A). PIV has been widely used to acquire kinematic information of cells in cell monolayers undergoing collective cell migration^32,33^. The validity of PIV analysis was tested by performing PIV analysis using synthetic movies as previously reported^34^. Synthetic movies were generated from fluorescence image of densely packed T cells by translating or rotating the snapshot image (Fig. 3B). PIV analysis results for the synthetic movies were compared with known displacements used to generate synthetic movies to assess error ranges. Translational velocity was estimated with <1% of errors, whereas rotational velocity was estimated with ~10% of errors (Fig. 3C) by PIV analysis.

### Analysis of T cell migration in microchannels densely packed with T cells

**Figure 4:**
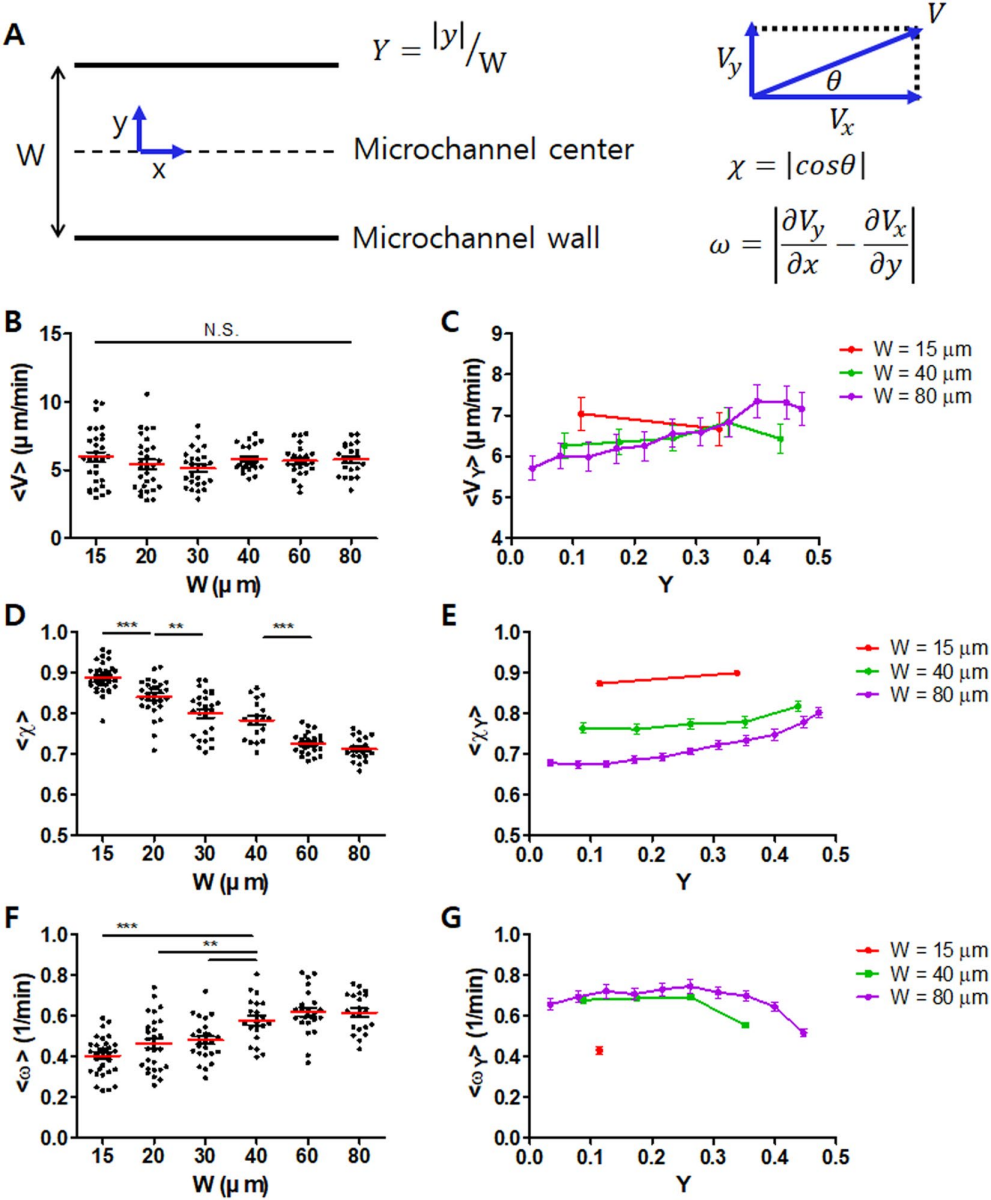
T cell motility in microchannels densely packed with T cells. (**A**) Schematic illustration of T cell motility parameters. (**B,C**) Time-averaged velocity of T cells in microchannels with various widths for entire positions 〈*V*〉 (**B**), and for normalized position Y 〈*V*_*Y*_〉 (**C**). (**D,E**) Time-averaged order parameter of T cells in microchannels with various widths for entire positions 〈*χ*〉 (**D**) and for normalized position Y 〈*χ*_*Y*_〉 (**E**). (**F,G**) Time-averaged vorticity of T cells in microchannels with various widths for entire positions 〈*ω*〉 (**F**), for normalized position Y 〈*ω*_*Y*_〉 (**G**). *n* = 32 for 15 μm, *n* = 29 for 20 μm, *n* = 26 for 30 μm, *n* = 20 for 40 μm, *n* = 24 for 60 μm, and *n* = 21 for 80 μm, Error bars (**C,E,G**) s.e.m. Statistical test (**B,D,F**) Mann-Whitney test, two-tailed, N.S.: not significant, **p < 0.01, ***p < 0.001. Data are representative of three independent experiments.

Time-lapse microscopy (10 min with 15 s interval) was conducted for microchannels densely packed with T cells with various widths, and then PIV analysis was performed for time-lapse images to assess motility of T cells in the microchannels. Importantly, we have used activated murine T cells in a resting phase that typically undergo minimal cell division (Fig. S1 in SI), and 10 min of observation time is too short to allow substantial cell division to happen during experiments, thus cell movement in PIV analysis is minimally affected by cell division. In narrow microchannels, densely packed T cells exhibited collective motion toward one direction along the microchannel (PIV image in Fig. 3A and Movie S1 in SI), whereas in wide microchannels, densely packed T cells appeared to migrate randomly, generating local swirls (PIV image in Fig. 3A and Movie S2 in SI). Motility of densely packed T cells in microchannels was quantitatively analyzed based on the coordinate system and parameters depicted in Fig. 4A using PIV data. Velocity *V*, which represents local translational motion, order parameter *χ*, absolute fraction of x-component velocity meaning the influence of microchannel walls on the migration direction of T cells, and vorticity *ω*, which represents local rotational motion, were calculated. For each parameter *Q* (*V*, *χ*, or *ω*), time-averaged value for entire microchannels 〈*Q*〉 was assessed by averaging values in all the pixels (3.5 μm × 3.5 μm) (Fig. 4B,D,F). Position dependent time-averaged value $\langle {Q}_{y}\rangle$ was calculated by averaging values of the pixels in particular position y, defined by the distance from the microchannel center (Fig. 4A). Then, positions of microchannels in different channel widths were normalized with channel width Y to obtain $\langle {Q}_{Y}\rangle$. Position dependent values for W = 15, 40 and 80 μm were combined and presented in Fig. 4C,E,G to show trends on how microchannel width affected each value, and the rest of the data (W = 20, 30, and 60 μm) were shown in Fig. S2 in SI.

Overall, densely packed T cells within the microchannels exhibited 〈*V*〉 ~ 7 μm/min, comparable to the velocity of T cells in lymph nodes^8^. 〈*V*〉 values of T cells in microchannels with various widths were not significantly different from each other (Fig. 4B), whereas position-dependent velocity profiles depended on the microchannel width (Fig. 4C). In narrow microchannels (W = 15~40 μm), relatively ‘flat’ velocity profile 〈*V*_*Y*_〉, or position-independent T cell velocity, was observed. In sharp contrast, T cells in wide mirochannels (W = 60 and 80 μm) exhibited position-dependent 〈*V*_*Y*_〉; T cells close to the microchannel walls (Y ~ 0.5) were significantly faster than T cells in central regions of microchannels (Y ~ 0). 〈*χ*〉 values gradually decreased as the microchannel width increased (Fig. 4D). 〈*χ*_*Y*_〉 exhibited similar trends to 〈*V*_*Y*_〉 (Fig. 4E); for narrow microchannels, it was near constant for all positions, whereas for wide microchannels, it was highest near microchannel walls (Y ~ 0.5) and lowest near the microchannel center (Y ~ 0). 〈*ω*〉 gradually increased as microchannel width increases (Fig. 4F). Due to limited resolution in PIV, a single vorticity value of 〈*ω*_*Y*_〉 was obtained for microchannels with W = 15 μm (Fig. 4G). For other microchannels (W = 20~80 μm), 〈*ω*_*Y*_〉 values were nearly constant except near microchannel walls. 〈*ω*_*Y*_〉 values near microchannel wall was the lowest, and close to that of microchannels with W = 15 μm.

These kinematic parameters represent distinct motility behaviors of T cells in microchannels with various widths. T cells in microchannels with W = 15 μm migrated to the direction nearly parallel to microchannel walls with minimal vorticity. Considering W = 15 μm < 2 × T cell diameter, most T cells were likely to be in contact with either of microchannel wall, thus their motility was highly influenced by the microchannel walls. Indeed, 〈*χ*_*Y*_〉 ~ 0.9, means T cells within microchannels mostly migrate toward the microchannel wall direction. T cells in microchannels with W = 40 μm mostly exhibited position independent motility behaviors except for the reduced vorticity near microchannel walls. In this case, W ~ 4 × T cell diameter, thus fraction of T cells contacting microchannels will be comparable to that of T cells non-contacting microchannels. 〈*V*_*Y*_〉 and 〈*χ*_*Y*_〉 values were nearly flat, but 〈*χ*_*Y*_〉 ~ 0.8, significantly lower than that of W = 15 μm, thus cell-cell interactions among neighboring T cells compensate microchannel wall effects and induce rather uniform T cell motility within the microchannels. Lastly, T cells in microchannels with W = 80 μm exhibited position dependent motility that velocity and order parameter gradually increased from center to wall, and vorticity decreased near the wall. Increased velocity near microchannel walls is likely due to guided protrusion and reduced collision. Microchannel wall effects mostly vanished within ~2 cell diameters, indicating short-range interactions among migrating T cells. Indeed, T cells only briefly interact with other T cells in lymph nodes in steady-state^35^, confirming weak and transient cell-cell interactions among migrating T cells resulting in minimal force propagation.

### Morphodynamics and motility of T cells in microchannels densely packed with T cells under various tonicity conditions

Next, we attempted to investigate roles of cytoskeletons and their regulators in T cell motility in densely packed microchannels using panels of pharmacological inhibitors. However, it was technically challenging to treat T cells in the central regions of microchannels with sufficiently high concentration of inhibitors because most of the inhibitors were absorbed by T cells near entry regions of microchannels, resulting in inhomogeneous distribution of inhibitors across microchannels. To avoid this issue, we pre-treated various pharmacological inhibitors (latrunculin A, blebbistatin, nocodazole, and taxol) prior to T cell loading, but in all cases, inhibitor-treated T cells failed to fill the microchannels with high density (Fig. S3 in SI). In contrast to pharmacological inhibitors that strongly bind to T cells, media osmolality that regulates membrane tension^36–38^ can be adjusted throughout the microchannels by the addition of DI water or sucrose. Osmolality of the media in the microchannels was adjusted by adding DI water or sucrose to the reservoir to the final osmolality of 140 mOsmol or 420 mOsmol, respectively. The osmolality of normal culture medium used in our experiments is 280 mOsmol. Tonicity conditions used in our experiments did not affect viability of T cells (Fig. S4 in SI). Since testing three different tonicity conditions to all microchannel widths was technically challenging, microchannels with W = 15 μm, in which T cells exhibit position independent kinematic behaviors, and 60 μm, in which T cells exhibit position dependent kinematic behaviors, were used for experiments.

**Figure 5:**
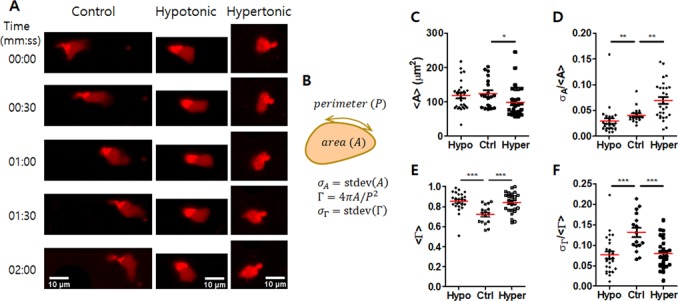
Morphology and morpholodynamics of densely packed T cells under tonicity variation. (**A**) Time-lapse images of T cells in microchannels (W = 60 μm) densely packed with T cells under various tonicity. To track individual T cells, ~5% of T cells were labeled with CellTrace Far Red, and time-lapse fluorescence images were acquired with 5 s intervals for 8 min. (**B**) Schematic illustration of quantitative assessment of T cell morphology and morphodynamics. (**C,D**) Effects of media tonicity on T cell area 〈*A*〉 (**C**), and area fluctuation over time ${\sigma }_{A}/\langle A\rangle$ (**D**). (**E,F**) Effects of media tonicity on T cell circularity 〈Γ〉 (**E**), and circularity fluctuation over time ${\sigma }_{{\rm{\Gamma }}}/\langle {\rm{\Gamma }}\rangle$ (**F**). *n* = 26 for Hypo, *n* = 16 for Ctrl, and *n* = 27 for Hyper. Statistical test: Mann-Whitney test, two-tailed, *p < 0.05, **p < 0.01, ***p < 0.001. Data are representative of three independent experiments.

Membrane tension is an important regulator of cell polarity and leading edge protrusion^39–41^, thus variation in media tonicity is likely to affect morphodynamics of T cells. To monitor morphodynamics of individual T cells in microchannels densely packed with T cells, ~5% of T cells was labeled with cytoplasmic dye CellTrace Far Red prior to seeding, and time-lapse imaging was conducted for 8 min with 5 s interval (Fig. 5A and Movies S3–5 in SI). Time-lapse images of each T cell was quantitatively analyzed to extract morphology and morphodynamic information of individual T cells (Fig. 5B): first, area (A) and circularity (Γ) of each T cell at every time point were measured. Then, time averaged value 〈*Q*〉 and relative standard deviation with respect to time ${\sigma }_{Q}/\langle Q\rangle$, which represents the relative magnitude of fluctuation over time, for each T cell in different experimental conditions were calculated and plotted for A and Γ (Fig. 5C–F). T cells in normal culture media (control) exhibited elongated morphology with extensive shape change (Fig. 5A and Movie S3 in SI), characteristics of amoeboid cell migration^13,14,42^. Alteration in media tonicity led to changes in T cell morphology and morphodynamics (Fig. 5A and Movies S4 and S5 in SI). T cells in hypotonic media exhibited rounded morphology, or significantly higher circularity than T cells in normal media (Fig. 5E), with minimal fluctuation of area and circularity (Fig. 5D,F). These results indicate that membrane tension increase by water incorporation in hypotonic media restrict membrane extension-mediated dynamic shape change of T cells. T cells in hypertonic media exhibited slightly decreased area (Fig. 5C) and increased circularity (Fig. 5E) compared with T cells in normal media, indicating reduced membrane tension by water loss caused shrinkage and rounding. In addition to the morphology changes, hypertonic media also caused enhanced area fluctuation (Fig. 5D) with reduced circularity fluctuation (Fig. 5F), indicating decreased membrane tension allowed extensive isotropic membrane extension with minimal shape changes.

**Figure 6:**
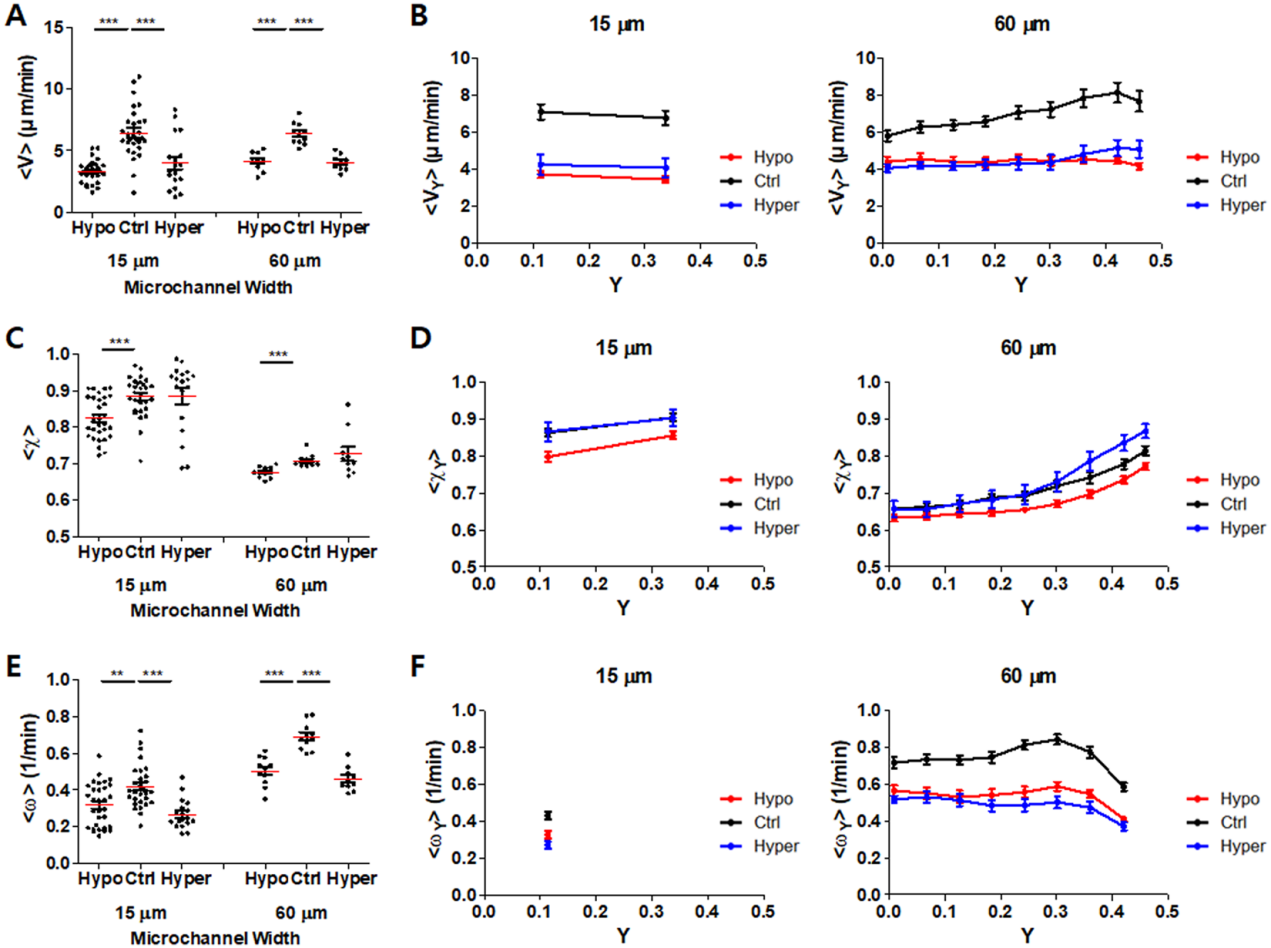
Effects of media tonicity on T cell motility in microchannels densely packed with T cells. (**A**,**B**) Effects of media tonicity on 〈*V*〉 (**A**), and 〈*V*_*Y*_〉 (**B**). (**C,D**) Effects of media tonicity on 〈*χ*〉 (**C**) and 〈*χ*_*Y*_〉 (**D**). (**E,F**) Effects of media tonicity on 〈*ω*〉 (**E**) and 〈*ω*_*Y*_〉 (**F**). 15 μm: *n* = 31 for Hypo, *n* = 29 for Ctrl, and *n* = 18 for Hyper; 60 μm: *n* = 11 for Hypo, *n* = 11 for Ctrl, and *n* = 10 for Hyper. Error bars (**B,D**,**F**) s.e.m. Statistical test (**A,C,E**) Mann-Whitney test, two-tailed, **p < 0.01, ***p < 0.001. Data are representative of three independent experiments.

With these characteristics in morphodynamics, we next examined the effects of media tonicity, or membrane tension, on T cell motility in microchannels densely packed with T cells. Time-lapse imaging was conducted, and PIV analysis was performed using time-lapse images to extract velocity field information (Movies S6~9 in SI). Using velocity field information, kinematic parameters described in Fig. 4A were further calculated and plotted (Fig. 6). Overall, changes in tonicity, either hypotonic or hypertonic, significantly reduced velocity and vorticity of T cells in microchannels regardless of W and positions (Fig. 5A,B,E,F). These results mean optimal membrane tension that facilitated amoeboid migration by extensive shape change is critical for rapid motility of T cells in T cell dense microenvironments. Of note, PIV analysis and single cell tracking (SCT) using ~5% T cells labeled with CellTrace Far Red exhibited identical trends, while mean velocity obtained by PIV analysis is consistently ~20% lower than that measured by SCT, similar to the previous PIV analysis^32^, further confirming validity of our PIV analysis (Fig. S5 in SI). Interestingly, 〈*χ*_*Y*_〉 values of T cells near microchannel walls, or Y ~ 0.5, in microchannels with W = 60 μm were significantly higher under hypertonic media condition than normal media condition (Fig. 6D). We speculate that extensive isotropic membrane extension of T cells under hypertonic condition (Fig. 5D,F) enhanced highly guided migration, or high 〈*χ*_*Y*_〉 values, of T cells near microchannel walls.

## Conclusion

In summary, we developed a technology that enabled quantitative assessment of T cell motility in T cell dense microenvironments, which mimic secondary lymphoid organs such as spleens and lymph nodes where T cell antigen recognition occurs. Microchannels densely packed with T cells were constructed by using trapezoid-shaped reservoirs guiding sedimentation of T cells near microchannel entries, and optimizing filling time and microchannel surface coating. Velocity field of T cells in microchannels densely packed with T cells was measured by PIV analysis, and other motility parameters, including order parameter and vorticity, were further extracted to assess the effect of microchannel dimensions and media tonicity. Our analysis revealed that weak interactions among neighboring T cells and dynamic polarization with extensive shape change were likely to be two key characteristics determining T cell motility in confined cell dense microenvironments. These results will provide new insights into immune cell migration in cell dense microenvironments.

## Methods

### Cell culture

DO11.10 T cell receptor transgenic mice (Jackson Laboratories) were bred in the POSTECH Biotech Center (PBC). All experiments involving mice, including euthanasia of mice and cell isolation, were performed in accordance with guidelines and regulations approved by the Institutional Animal Care and Use Committee at PBC. DO11.10 T cells were harvested from spleens and lymph nodes of DO11.10 mice and stimulated with 1 μg/ml of OVA323-339 peptide (ISQAVHAAHAEINEAGR, Peptron, Korea). DO11.10 T cells were grown in R10 media, RPMI 1640 media (Invitrogen) supplemented with 10% FBS (Gibco), 100 U/ml penicillin, 100 mg/ml streptomycin (Invitrogen), and 0.1% beta-mercaptoethanol (Sigma). On day 2 of stimulation, 1 U/ml of IL-2 was added and cells on day 5 were used for experiments.

### Microchannel fabrication

Poly (dimethyl siloxane) (PDMS) microchannels were fabricated by following the procedure described below. First, photolithography was performed on silicon wafers using positive photoresists AZ 4533 (MicroChemicals, Germany) and AZ ®826 MIF (MicroChemicals, Germany) developers. Then, the patterned wafer was etched using a deep reactive ion etcher (DRIE, STS) until a depth of 4 μm was reached. The etched wafer was sonicated in acetone to remove remaining PR and clean the wafer. Micropatterns on Si wafer was replicated to the PET film using poly(urethane acrylate) (PUA)-based capillary force lithography^43,44^. PDMS precursor solution (10:1 ratio of base and curing agent of SYLGARD 184; Dow Corning) was poured on the patterned PET film and cured to obtain PDMS microchannels. Then, trapezoid-shaped reservoirs were made at both end of microchannels by handcraft using a razor blade. To attach PDMS microchannels on glass coverslips, the PDMS microchannels were treated with air plasma (100 W, Femto Science, Korea) for 60 s, placed on clean glass coverslips, and baked using a 120 °C hot plate for 3 min.

### T cell filling into microchannel

The microchannels was coated with 10 μg/ml of ICAM-1 (R&D Systems), 1% BSA (Sigma-Aldrich) or 0.2% pluronic F-127 (Thermo Fisher Scientific), dissolved in PBS. Then, the solution-filled PDMS microchannels was incubated at 37 °C for 90 min. After washing with PBS 3 times, T cells in R10 media (2 × 10^6^ cells/200 μl) were placed in a reservoir of the microchannel and incubated in a CO_2_ controlled incubator at 37 °C.

To evaluate packing density of T cells, T cells labeled with 10 μg/ml Hoechst 33342 (Life Technology) were seeded into the microchannels coated with ICAM-1, BSA or pluronic F-127. After 3, 6, 9, and 12 h of seeding, T cell nucleus images were acquired to calculate packing density.

### Migration assay

A modified Zeiss Axio Observer.Z1 epifluorescence microscope with a X40 (Plan-Neofluar, NA = 1.3) objective lens and a Roper Scientific CoolSnap HQ CCD camera was used for imaging. XBO 75 W/2 Xenon lamp (75 W, Osram) and DAPI (EX 365, BS 395, EM BP 445/50), Cy3 (EX BP 550/25, BS 570, EM BP 605/70), Cy5 (EX BP 620/60, BS 660, EM BP 770/75) filter sets were used for fluorescence imaging. The microscope was automatically controlled using Axiovision 4.6 (Carl Zeiss). Microchannels densely packed with T cells were mounted on a microscope stage equipped with a Chamlide TC incubator system (maintaining 37 °C and 5% CO_2_). Migration of densely packed T cells in microchannels was recorded by video microscopy with 15 s interval for 10 min. T cells within 400 μm of the entry and exit microchannels were excluded to avoid entry/exit effects. Time-lapse imaging was performed after seeding T cells in reservoirs and incubating for 9 h, sufficient time to equilibrate pressure differences between two reservoirs. Since temporal pressure gradients can arise for many reasons, including media evaporation, and such pressure gradients can induce convective flow in microchannels that can influence cells^45,46^, we carefully monitored whether we had unidirectional T cell migration mediated by convection in each experiment. Indeed, we have not observed any bulk convective motion of T cells in microchannels.

For PIV analysis, T cells were stained with 0.3% vybrant DiI cell labeling solution (Molecular Probes) for 15 min. For morphodynamics measurement, ~5% of T cells were stained with 1 μM CellTrace Far Red for 20 min. For tonicity variation, media in each reservoir was changed to hypertonic (R10 media containing 140 mM sucrose) or hypotonic (DI water: R10 media = 1:1) media for 1.5 h prior to time-lapse imaging.

### Motility analysis based on PIV

Time-lapse images were processed and analyzed using Matlab R2017a (MathWorks) and Image J (NIH). PIV analysis was performed using the Matpiv software package^47^, using 50% overlap and 16 pixels of final interrogation window. The peaks with low signal-to-noise ratio were filtered out, and the deleted points were inserted using nearest neighbor interpolation. *V*, *χ*, and *ω* were calculated using velocity field information obtained by PIV analysis.

To validate PIV analysis, synthetic movies with known displacements were generated and analyzed with PIV as previously described^34^. The synthetic movies were made from snapshot of 0.3% vybrant DiI labeled T cells by translating the snapshot 5 pix/frame or rotating the snapshot 1 degree/frame clockwise. The acquired PIV results were compared with known displacements of synthetic movies and errors were calculated.

### Morphology and morphodynamics analysis

Morphological analysis of T cells was performed using Matlab custom code. From the original fluorescence image, a region containing a T cell of interest was manually cropped and imported to Matlab for further processing (Fig. S6 in SI). After filtering the image using ‘median’ filter to reduce shot noise (Fig. S6B), the filtered image was converted to a binary image (Fig. S6C), and processed by ‘morphological close’. Then, area (*A*), perimeter (*P*), center of mass and circularity (${\rm{\Gamma }}=4{\rm{\pi }}A/{P}^{2}$), of individual T cells were calculated from the filtered images. Area fluctuation over time, or ${\sigma }_{A}/\langle A\rangle$, was calculated as the ratio of standard deviation of area and time-averaged area of individual T cells, and circularity fluctuation over time, or ${\sigma }_{{\rm{\Gamma }}}/\langle {\rm{\Gamma }}\rangle ,$ was calculated as the ratio of standard deviation of circularity and time-averaged circularity of individual T cells.

### Statistical analysis

Statistical significance was tested using the Mann-Whitney U-test. For bar graphs, average values with standard error of mean (s.e.m.) are presented. All statistical analysis were performed using Prism (GraphPad Software).

## Supplementary information

Supplementary Information

Supplementary Movie S1

Supplementary Movie S2

Supplementary Movie S3

Supplementary Movie S4

Supplementary Movie S5

Supplementary Movie S6

Supplementary Movie S7

Supplementary Movie S8

Supplementary Movie S9

## Data Availability

The datasets generated and analyzed during the current study are available from the corresponding author on reasonable request.
