# Supplementary material for: T cell migration in microchannels densely packed with T cells
Source: Sci Rep. 2019 May 10;9:7198. doi: 10.1038/s41598-019-43569-w (PMC6510777; doi:10.1038/s41598-019-43569-w)
Supplement: Supplementary file 1 — Supplementary Information [file 41598_2019_43569_MOESM1_ESM.pdf]

T cell migration in microchannels densely packed with T cells

HyoungJun Park ,[a] Junsang Doh[*,a,b,c]*

[a]Department of Mechanical Engineering, [b]School of Interdisciplinary Bioscience and Bioengineering (I-Bio), Pohang University of Science and Technology (POSTECH) San 31, Hyoja-dong, Nam-Gu, Pohang, Gyeongbuk, 790-784, Korea. [c]Department of Materials Science and Engineering, Seoul National University, 1 Gwanak-ro, Gwanak-gu, Seoul 08826, South Korea

[*]Corresponding author:

Junsang Doh

Address: Seoul National University, 1 Gwanak-ro, Gwanak-gu, Seoul 08826, South Korea

Tel: +82-2-880-1605

Fax: +82-2-885-9671

E-mail: jsdoh@snu.ac.kr

Supplementary Information (SI)

Supplementary movie legends

Movie S1. Representative DiI fluorescence/velocity vector overlay movie of 15 μm width microchannels densely packed with T cells. Scale bar: 15 μm. Elapsed time: mm:ss.

Movie S2. Representative DiI fluorescence/velocity vector overlay movie of 60 μm width microchannels densely packed with T cells. Scale bar: 15 μm. Elapsed time: mm:ss.

Movie S3. Representative CellTrace Far Red fluorescence movie of T cell in 60 μm width microchannels densely packed with T cells in control media. Scale bar: 10 μm. Elapsed time: mm:ss.

Movie S4. Representative CellTrace Far Red fluorescence movie of T cell in 60 μm width microchannels densely packed with T cells in hypotonic media. Scale bar: 10 μm. Elapsed time: mm:ss.

Movie S5. Representative CellTrace Far Red fluorescence movie of T cell in 60 μm width microchannels densely packed with T cells in hypotonic media. Scale bar: 10 μm. Elapsed time: mm:ss.

Movie S6. Representative DiI fluorescence/velocity vector overlay movie of 15 μm width microchannels densely packed with T cells in hypotonic media. Scale bar: 15 μm. Elapsed time: mm:ss.

Movie S7. Representative DiI fluorescence/velocity vector overlay movie of 60 μm width microchannels densely packed with T cells in hypotonic media. Scale bar: 15 μm. Elapsed time: mm:ss.

Movie S8. Representative DiI fluorescence/velocity vector overlay movie of 15 μm width microchannels densely packed with T cells in hypertonic media. Scale bar: 15 μm. Elapsed time: mm:ss.

Movie S9. Representative DiI fluorescence/velocity vector overlay movie of 60 μm width microchannels densely packed with T cells in hypertonic media. Scale bar: 15 μm. Elapsed time: mm:ss.
